# Diagnostic accuracy of ultrasonography and 99m-Technetium Sestamibi scintigraphy for the preoperative localization of Parathyroid Adenoma keeping histopathological findings as reference standard

**DOI:** 10.12669/pjms.40.10.8575

**Published:** 2024-11

**Authors:** Tahir Ghaffar, Shaista Kanwal, Azizul Hasan Aamir, Nizamud Din

**Affiliations:** 1 Tahir Ghaffar, MBBS, FCPS, MRCP, Department of Diabetes, Endocrinology and Metabolic Diseases, MTI Hayatabad Medical Complex, Peshawar, Pakistan; 2 Shaista Kanwal, MBBS, FCPS, MRCP, Department of Diabetes, Endocrinology and Metabolic Diseases, MTI Hayatabad Medical Complex, Peshawar, Pakistan; 3 Azizul Hasan Aamir, MRCP, FRCP, FACE, Department of Diabetes, Endocrinology and Metabolic Diseases, MTI Hayatabad Medical Complex, Peshawar, Pakistan; 4 Nizamud Din, MBBS, FCPS, Department of Diabetes, Endocrinology and Metabolic Diseases, MTI Hayatabad Medical Complex, Peshawar, Pakistan

**Keywords:** Primary Hyperparathyroidism, Parathyroid Ultrasound, 99m-Tc Sestamibi Scintigraphy

## Abstract

**Objective:**

To determine the diagnostic accuracy of Ultrasound (US) and 99m-Tc Sestamibi scan in patients with primary hyperparathyroidism (PHPT) for the localization of parathyroid adenoma before surgery keeping Parathyroid histopathology as reference standard

**Method:**

This three years retrospective study was performed in the Department of Endocrinology, Hayatabad Medical Complex, Peshawar. Patients with PHPT who underwent parathyroidectomy from July 2019 to June 2022 were included in the study. Information relating to localization studies prior to surgical management like US and 99m-Tc Sestamibi scan was documented. These imaging findings were subsequently compared with the findings of parathyroid surgery and histopathology results which were taken as reference standard.

**Results:**

The sensitivity, positive predictive value (PPV) and overall accuracy of US for the preoperative localization of parathyroid adenoma was 77%, 95.7% and 75%, respectively. Whereas the sensitivity, PPV and accuracy of 99m-Tc Sestamibi scintigraphy was 89.5%, 95% and 86% respectively.

**Conclusion:**

Ultrasound despite its cost effectiveness has a lower sensitivity compared to 99m-Tc Sestamibi scintigraphy. Similarly, the sensitivity and overall accuracy of US and 99m-Tc Sestamibi scan when taken in combination is higher compared to either modality. It is thus recommended that the combination of these modalities should be employed to localize the adenomas accurately for surgery of the parathyroid gland for a better outcome.

## INTRODUCTION

Primary hyperparathyroidism (PHPT) is a common endocrine disorder characterized by the increased secretion of parathyroid hormone leading to hypercalcemia.^1^ With the introduction of the auto analyzer for routine screening of serum calcium, there has been a great change in the clinical spectrum of PHPT over the last few years with the disease being diagnosed at a younger age and at an asymptomatic stage. The introduction of intact PTH (iPTH) has further narrowed down the differentials of the origin of the disease.^2^ The age adjusted prevalence of PHPT in 2010 in the United States is reported to be 233 per 100,000 in females and 85 per 100,000 in males. Whereas it was stated to be 0.2% in the middle aged and elderly Chinese.^3^ The commonest underlying cause of the disease is a single parathyroid adenoma reported in 80 to 85 % cases followed by multi gland disease seen in approximately 5% cases, with parathyroid hyperplasia and carcinoma seen much less frequently.^4^

The definitive treatment for PHPT is surgical resection.^5^ Previously, open neck surgery was employed for the surgical removal of the parathyroid gland. But, over the past couple of years, minimally invasive parathyroidectomy (MIP) is adopted to make surgeries less invasive and with reduced number of perioperative and postoperative complications.^6^ It is of utmost importance to accurately localize the abnormal parathyroid glands before surgery. Failure to localize the parathyroid adenoma accurately would lead to failed and prolonged surgery with increased risk of complications and greater chance of re exploration.^7^ Several imaging techniques are utilized for the localization of parathyroid adenomas before surgery. These include ultrasonography (US), dual phase 99m-Tc sestamibi scan, four dimensional CT scan and MRI.^8^ Newer technologies like 99m-Tc sestamibi scintigraphy in combination with single-photon emission CT (SPECT/CT) and positron emission tomography (PET)/CT are employed in case of inconclusive results on initial imaging.^9^ Amongst these, US and 99m-Tc sestamibi scan are widely used with sensitivity ranging from 80 to 94.4% for US and 83 to 97.4% for Sestamibi, while specificities for US and sestamibi scan ranging from 44.4 to 77% and 71.4 to 87%, respectively.^10^

In Pakistan, very few studies have explored the diagnostic performance of US and Sestamibi scan for parathyroid adenoma localization.^11^,^12^ Preoperative localization of parathyroids using US and 99m-Tc sestamibi was examined in only one study from Pakistan, and the combined accuracy of both modalities for localization was reported as 100%.^12^ However additional studies are required in this regard keeping in mind that Pakistan is a low-income country where resources are inadequate, and modern techniques are not only costly but difficult to access as well. Thus, the objective of this study was to determine the diagnostic accuracy of Ultrasound and 99m-Tc Sestamibi scan for the localization of parathyroid adenoma before surgery keeping Parathyroid histopathology as reference standard.

## METHODS

This was a three years retrospective study, performed in the Department of Endocrinology, Hayatabad Medical Complex Peshawar.Information of patients with PHPT was retrieved from the hospital files from 1^st^ July 2019 to 30^th^ June 2022. The study comprised of patients 18 years and beyond of either gender with PHPT who had undergone parathyroid surgery. The identity of the study patients was kept anonymous to maintain confidentiality. Patients with increased serum calcium levels and patients with normal serum calcium levels with increased or inappropriately normal levels of PTH were diagnosed to have PHPT. Those patients who had increased calcium levels due to either secondary or tertiary hyperparathyroidism, and those with hypercalcemia due to malignancy other than parathyroid

### Ethical Approval:

It was obtained from the ethical committee of the hospital (Approval number 1342, date September 6, 2022).

Information such as patient demographics, clinical manifestations, symptoms leading to the diagnosis and laboratory parameters at presentation like serum calcium, phosphorus, alkaline phosphatase, serum albumin, renal function tests, vitamin-D levels and serum PTH were recorded. Serum calcium was corrected for the respective albumin level. Information relating to localization studies prior to surgical management like US using 10-12 hertz high-frequency linear probe and 99m-Tc Sestamibi scan was documented. These imaging findings were subsequently compared with the findings of parathyroid surgery and histopathology results. The findings of the histopathology result were taken as reference standard. Ultrasound with a high-frequency (5-10 MHz) probe was performed by an experienced Radiologist with at least five years’ experience at the department of Radiology, Hayatabad medical complex, Peshawar. 99m-Tc Sestamibi scan was conducted by an experienced Nuclear Physician with a minimum five years’ experience at Institute of Radiotherapy and Nuclear Medicine, IRNUM, Peshawar.

Cases where an abnormality of parathyroid gland was detected and corresponded to the histological findings were referred to as True positive cases (TP), whereas events where abnormality detected on imaging were not compatible with the histological findings were deemed false positive (FP). Negative imaging findings detected on histology were labelled as false negative (FN) while abnormality not being detected on imaging as well as on surgery were considered as true negative (TN). The diagnostic accuracy of each imaging modality was assessed by assessing sensitivity, specificity, positive predictive value (PPV), negative predictive value (NPV) and overall accuracy of each imaging technique. These were then compared with the findings of the histopathology result which were considered as gold standard. The statistical program, SPSS version 20 was utilized for statistical analysis.

## RESULTS

A total of 59 patients with PHPT were selected, who had undergone parathyroid surgery from 1^st^ July 2020 to 30th June 2022. The mean age of the study participants was 38.36 ± 13.55 years. There were 33 (55.9%) males and 26 (44.1%) females out of the total 59 patients. The baseline laboratory parameters of the study participants are presented in Table-I. Preoperative US and 99m-Tc Sestamibi scan was accomplished in all patients. All the patients underwent histological evaluation of the surgical specimen. Fifty-seven (96.6%) out of fifty-nine patients were found to have parathyroid adenoma. The remaining two patients had thyroid tissue on histology.

**Table-I:** Baseline Laboratory parameters of patients.

Variables	Patients (n=59)
Age (years)	38.36±13.55
Calcium (mg)	12.88± 1.31
Phosphorus (mg)	2.45± 0.78
Alkaline Phosphatase	534.50±500.92
Creatinine (mg/dl)	0.98±0.37
24-hour urinary calcium (mg/24 hours)	369.33±106.74
Vitamin-D (ng/ml)	29.93± 7.94
Parathyroid Hormone (PTH) (pg/ml)	935.96±765.60

The true positives, false positives and false negative cases on US were 44 (74.6%), 2 (3.4%) and 13 (22.03%), respectively. On the other hand, true positive, false positive and false negative cases on 99m-Tc Sestamibi scan were 51 (86.4%), 2 (3.4%) and 6 (10.2%), respectively. These are presented in Table-II. The sensitivity of US was 77% while that of 99m-Tc Sestamibi scan was 90%. The positive predictive value of both modalities was 96%. Overall Accuracy of US was 75% while that of Sestamibi scintigraphy was 86%. These are presented in Table-III. The specificity and negative predictive values were not measured as there were no true negative cases in either imaging techniques.

**Table-II:** True positive, true negative, false positive and false negative cases for Ultrasonography and 99m-Tc Sestamibi Scan.

Imaging Technique	True Positive	True Negative	False Positive	False Negative
Ultrasonography of Neck (n=59)	44	13	2 (Both cases were multinodular goiter)	13
99m-Tc Sestamibi scan (n=59)	51	06	2 (Both cases were multinodular goiter)	06

**Table-III:** Diagnostic accuracy of Ultrasonography and 99m-Tc Sestamibi Scan.

Imaging modality	Sensitivity	Positive predictive value	Accuracy
Ultrasonography of Neck	77%	95.7%	75%
99m-Tc Sestamibi scan	89.5%	96%	86%
Combined US and Sestamibi scan	89.5%	96.2%	89.8%

***Note:*** The specificity and negative predictive value were not measured as there were no true negative cases on either imaging techniques.

## DISCUSSION

The current study revealed higher sensitivity and overall accuracy of 99m-Tc Sestamibi scintigraphy compared to US. It also revealed that combined US and 99m-Tc Sestamibi scan had higher sensitivity and overall accuracy compared to either modality. An exact localization of the parathyroid adenoma before surgery is mandatory for successful parathyroid surgery. US and Tc-99m Sestamibi scan are the two widely used modalities employed for this purpose with satisfactory sensitivity and specificity.^10^ The present study revealed that US has a sensitivity of 77 % while PPV of 96 % whereas Tc 99m Sestamibi scan had a sensitivity of 90 % and PPV of 96 % for localization of parathyroid adenoma. These are comparable to the findings of a meta-analysis which stated a pooled sensitivity and PPV of US to be 76.1 % and 93.2 %, respectively.^13^ A study conducted in UK revealed sensitivity of US to be 70 % with overall accuracy of 68 %.^14^

Another study revealed sensitivity of US to be 56.7 % while PPV of 96.3%.^15^ The sensitivity of this study was much lower compared to our study while the PPV was comparable to that of our study. Another study revealed 76 % sensitivity of US for preop localization of parathyroid adenoma.^16^ The sensitivity range of US has been reported to be between 44%-90%.^17^

In Pakistan, only limited studies have been conducted regarding the validity of different imaging techniques for parathyroid adenoma localization.^11^,^12^ A study conducted in Pakistan showed a combined accuracy of 99m-Tc Sestamibi scan and US to be 100 % for parathyroid gland localization however this study did not evaluate the individual sensitivity and specificity of each modality.^12^ A study conducted in China revealed sensitivity of US to be 90.70%.^18^

Ultrasound is an inexpensive, easily available investigation and without exposure to ionizing radiation.^19^ However, there are several factors which has led to the huge difference in the sensitivity and specificity profile of US between these different studies. Firstly, US is greatly dependent on the experience of the Sonologist. Secondly, the recent advancement in the US technology might have resulted in these differences.^17^ Finally, the results are also dependent upon other factors like the location of the parathyroid adenomas, with lesions located in the retroesophageal area, mediastinum and those located deeper, and smaller adenomas pose difficulty in localizing.^19^

In the current study, out of 59 cases, 13 cases of parathyroid adenoma were not detected by the US, out of which five cases were that of ectopic parathyroid adenomas. Out of six false negative cases of 99M-Tc Sestamibi scan, four were ectopically located glands. This means that more than 50 % of the adenomas were detected on the sestamibi scan. Pakistan being an underdeveloped country, with very limited healthcare resources might be still lagging in the technological advancements of Ultrasonography. This factor and the expertise of the Radiologist in US of the parathyroid gland specifically could have resulted in the comparatively lower sensitivity of the US in the detection of the adenomas in the current study.

Traditionally, 99 m-Tc Sestamibi scans have been mandatory investigation of patients with PHPT before surgery.^17^ In the current study, the sensitivity of 99m-Tc Sestamibi scintigraphy is 90 % and PPV of 96 %. These results are comparable to that of a study by Ozkaya et al. which revealed sensitivity of Sestamibi scan to be 90.9%.^20^ In another study, the sensitivity of sestamibi was 93 %.^21^ Studies have shown that the sensitivity of Sestamibi scan ranges between 83% to 97.4%. A meta-analysis has showed pooled sensitivity and specificity for Sestamibi scan to be 83% and 87% respectively.^10^

In the present study the sensitivity of Sestamibi scan is much higher compared to that of US. However, globally due to the technological advancements of US and the increased expertise of the Sonologist in parathyroid imaging has yielded higher sensitivity and specificity profile of US. Studies have shown that the accuracy of US for adenoma localization is 93 % compared to 90% accuracy by Sestamibi scan. They have also demonstrated that the sensitivity of US is 98 % compared to Sestamibi which showed 93 % sensitivity.^21^

Our study revealed higher sensitivity of 99m-Tc Sestamibi scan for the parathyroid gland localization before surgery compared to US. This is because the US has several limitations as described previously. And Pakistan being an underdeveloped country is still lagging behind. Thus, it is postulated that though the US is highly sensitive but keeping in view the results of the current study, it is important to consider that accurate preoperative localization should be done before proceeding to surgery. Moreover, the present study has also revealed that the sensitivity of both imaging modalities when taken together is 89.5% with an overall accuracy of 89.8% which is greater than individual accuracy of either technique.

This has also been revealed in other studies as well. US is of importance in evaluating the anatomical details while Sestamibi scan is greater importance when it comes to providing details regarding ectopically located glands, smaller adenomas and the simultaneous presence of thyroid nodules. It also helps in the understanding of the results of parathyroid US. Thus, the combined use of both modalities is superior to the individual technique.^16^ Additionally, it is important to realize that the presence of multinodular thyroid disease leads to false positive on both imaging modalities. Therefore, thyroid nodular disease must be screened especially in areas of endemic goiter and one should not rely on US for the preoperative localization of parathyroid adenomas.

### Limitations:

The focus of this study on the limitations of US and the advantages of the 99m-Tc Sestamibi scan is especially pertinent to developing nations like Pakistan. The findings of this study emphasize the necessity of using advanced imaging methods to enhance preoperative planning and recommend that, even in situations with limited resources, attempts should be made to use more sensitive imaging modalities.

## CONCLUSION

Our study revealed higher sensitivity and overall accuracy of 99m-Tc Sestamibi scintigraphy compared to US. It also revealed higher sensitivity and overall accuracy of US and 99m-Tc Sestamibi scan when taken in combination compared to either modality. It is thus recommended that the combination of these modalities should be employed to localize the adenomas accurately for surgery of the parathyroid gland. This will reduce the number of false positive cases, improve the outcome of parathyroid surgery, and will reduce the morbidity and mortality of patients with PHPT.

### Author’s Contribution:

**TG:** Conceived, designed, did literature review, performed statistical analysis & drafted the manuscript.

**SK:** Participated in analysis, literature review and interpretation of data, and helped in drafting the manuscript.

**AHA:** Conceived, designed, and critically revised the manuscript.

**ND:** Participated in data collection, did literature review and interpretation of data.

All authors provided final approval for publication of the manuscript and are responsible for the integrity of the study.
